# The accuracy with which the 5 times sit-to-stand test, versus gait speed, can identify poor exercise tolerance in patients with COPD: A cross-sectional study

**DOI:** 10.1097/MD.0000000000004740

**Published:** 2016-09-02

**Authors:** Roberto Bernabeu-Mora, Francesc Medina-Mirapeix, Eduardo Llamazares-Herrán, Silvana Loana de Oliveira-Sousa, Mª Piedad Sánchez-Martinez, Pilar Escolar-Reina

**Affiliations:** a Division of Pneumology, Hospital Morales Meseguer; b Department of Physical Therapy, University of Murcia, Murcia; c Division of Rehabilitation, Hospital Ramón y Cajal, Madrid; d Department of Physical Therapy, Catholic University, Murcia, Spain.

**Keywords:** 4-m gait speed, 5-repetition sit-to-stand, 6-minute walk test, chronic obstructive pulmonary disease, Hospital Anxiety and Depression Scale, quadriceps muscle strength, Short Physical Performance Battery

## Abstract

Identifying those patients who underperform in the 6-minute walk test (6MWT <350 m), and the reasons for their poor performance, is a major concern in the management of chronic obstructive pulmonary disease.

To explore the accuracy and relevance of the 4-m gait-speed (4MGS) test, and the 5-repetition sit-to-stand (5STS) test, as diagnostic markers, and clinical determinants, of poor performance in the 6MWT.

We recruited 137 patients with stable chronic obstructive pulmonary disease to participate in our cross-sectional study. Patients completed the 4MGS and 5STS tests, with quantitative (in seconds) and qualitative ordinal data collected; the latter were categorized using a scale of 0 to 4. The following potential covariates and clinical determinants of poor 6MWT were collated: age, quadriceps muscle-strength (QMS), health status, dyspnea, depression, and airflow limitation. Area under the receiver-operating characteristic curve data (AUC) was used to assess accuracy, with logistic regression used to explore relevance as clinical determinants.

The AUCs generated using the 4MGS and 5STS tests were comparable, at 0.719 (95% confidence interval [CI] 0.629–0.809) and 0.711 (95% CI 0.613–0.809), respectively. With ordinal data, the 5STS test was most accurate (AUC of 0.732; 95% CI 0.645–0.819); the 4MGS test showed poor discriminatory power (AUC <0.7), although accuracy improved (0.726, 95% CI 0.637–0.816) when covariates were included. Unlike the 4MGS test, the 5STS test provided a significant clinical determinant of a poor 6MWT (odds ratio 1.23, 95% CI 1.05–1.44).

The 5STS test reliably predicts a poor 6MWT, especially when using ordinal data. Used alone, the 4MGS test is reliable when measured with continuous data.

## 1 Introduction

Exercise intolerance is an important indicator of the severity of chronic obstructive pulmonary disease (COPD)^[1]^; this disability erodes the ability to perform everyday tasks, and substantially diminishes quality of life.^[2,3]^ For this reason, our ability to quantify exercise capacity using tests such as the 6-minute walk test (6MWT) is a necessary and integral part of the routine clinical assessment of COPD to timely identify the functional decline, and to provide advice and preventive interventions.^[4]^

The 6-minute walk test (6MWT) is widely regarded to be a reliable measure of exercise tolerance in patients with COPD,^[5]^ and a good predictor of morbidity and mortality.^[6]^ Indeed, walking less than 350 m in this test has been associated with a significant increase in mortality.^[3]^ Given the prognostic significance of performing poorly on the 6MWT, some authors have gone on to identify degree of airflow limitation, dyspnea, and the presence of depressive symptoms as 3 significant clinical determinants of a poor performance in this test (<350 m).^[7]^

Despite the relevance of the 6MWT, it is not typically measured by pulmonologists in the clinic due to time constraints, equipment, and obvious space requirements.^[8]^ Instead, there is currently the tendency to identify tests and measures that are faster, and need less space, but still reliably measure exercise capacity.^[9,10]^ In this sense, the 4-m gait-speed (4MGS) test, or 5-repetition sit-to-stand (5STS) test are plausible substitutes for the 6MWT.^[8,10]^ Additionally, the 4MGS has been recently recommended as a potentially useful marker to classify subjects with poor 6MWT performance.^[11]^

Factors other than exercise tolerance can influence scores for these 2 tests in COPD patients (i.e., test scores tend to decline with increasing age).^[10,11]^ However, to date, it remains unknown as to which factors or covariates associated with the 4MGS test affects accuracy to classify subjects between a normal and poor 6MWT performance. Moreover, the capacity of the 5STS test to accurately report, and act as a clinical determinant of exercise intolerance, also remains largely untested.

The primary aim of this study was to identify whether, and to what extent, both the 4MGS and 5STS tests could be used as diagnostic markers, and clinical determinants, of a poor 6MWT performance. Our specific objectives were: to explore whether covariates associated with the 5STS and 4MGS tests could substantially affect their accuracy; their ability of these tests to be used as stand-alone markers of performance; and to explore their relative efficacy as clinical determinants of a poor 6MWT performance, after adjustment for other clinical determinants. We hypothesized that both tests, recorded using continuous data (seconds), or ordinal data (0–4) as used in the short physical performance battery (SPPB),^[12]^ would show good and comparable discriminatory accuracy, and that this accuracy would improve after correction for significant covariates.

## 2 Methods

### 2.1 Study design and participants

A controlled, cross-sectional study was devised. Patients with stable COPD were prospectively recruited from an outpatient pulmonary service at Meseguer Hospital in Murcia, Spain, during 2015. All study participants provided written informed consent, and the study protocol was approved by the institutional review board of the hospital called “Comité Ético de Investigación Clínica del Hospital General Universitario José María Morales Meseguer” (approval number: EST-35/13).

Inclusion criteria included COPD patients with a forced expiratory volume in 1 second (FEV_1_)/forced vital capacity ratio of <70% of the predicted value, and an age of between 40 and 80 years. The diagnosis of COPD, and its severity, was based of the Global Initiative for Chronic Obstructive Lung Disease (GOLD) guidelines.^[13]^ Patients with an unstable cardiac condition within 4 months of the start of the study were excluded, as were those with cognitive deterioration, or a limitation in walking. During a 1-year period, a consecutive sample of eligible patients was identified from patient health examinations. A pulmonary physician assessed the eligibility criteria for recruitment.

### 2.2 Measures

#### 2.2.1 Six-minute walk test

The 6MWT was performed indoors, along a flat, straight, 30-m walking course, supervised by 2 well-trained nurses (with a mean of 19 years of experience), according to American Thoracic Society guidelines.^[14]^ Patients were instructed and encouraged to walk as far as possible in 6 minutes, using standard incentive phrases every minute. Patients were permitted to stop and rest during the test, but were instructed to resume walking as soon as they felt able to do so.

#### 2.2.2 Physical performance tests and potential covariates

Participants performed the 4MGS test and the 5STS test according to the National Institute on Aging protocol for the SPPB.^[15]^ The 5STS measures the time (in seconds) taken to stand 5 times from a sitting position, as rapidly as possible. The 4MGS test measures the time taken to cross a 4-m line at usual speed. Categorical scores (range 0–4) for both the 4MGS and the 5STS tests were based on timed quartiles, established previously in a large population.^[12]^

A total of 5 variables were selected from our literature search, and measured as potential covariates, based on their association with either test. These variables were age (years), quadriceps muscle strength (QMS), perception of health status, dyspnea, and reporting of symptoms of depression.^[10,11,16]^ Most of these variables—the 5STS and the 4MGS tests—were measured by a well-trained pulmonology physician (12 years of experience). QMS was measured first, and then, the 4MGS and the 5STS tests were measured. Afterwards, the 6MWT was performed. Finally, self-reported questions were surveyed using specific questionnaires.

The QMS was measured using a calibrated dynamometer. Based on previous descriptions,^[17]^ participants remained seated on a raised plinth, with their assessor placing the participant's knee in a flexed position, at 70°. The dynamometer was placed perpendicular to the anterior tibia, 5 cm above the lateral malleolus, with force applied gradually over a 4-second period to allow maximal muscle fiber recruitment. Two trials per test were performed, as previously described.^[17]^

Perception of health status was assessed using the COPD Assessment Test (CAT).^[18]^ Dyspnea was measured by the self-administered modified Medical Research Council (mMRC) dyspnea scale.^[19]^ Presence of depressive symptoms were assessed according to the Hospital Anxiety and Depression Scale (HADS).^[20]^ This scale ranges from 0 to 21 points. Binary categories (yes/no) were created for this study, using an accepted cut-off of 8 for depression.^[21]^ QMS was measured first, and then, the 4MGS and the 5STS tests were measured. Afterwards, the 6MWT was performed. Finally, self-reported questions were surveyed using specific questionnaires.

#### 2.2.3 Clinical determinants of a poor 6MWT performance

We selected 3 significant clinical determinants of a poor 6MWT performance (<350 m) as determined by Spruit et al.^[7]^ These were airflow limitation by GOLD guidelines^[13]^, dyspnea, and symptoms of depression. Airflow was measured using a MasterScope spirometer. To calculate the per cent of predicted pulmonary function, we used predictive equations derived from the Third National Health and Nutrition Examination Survey.^[22]^

### 2.3 Statistical analyses

Descriptive statistics were used to characterize our patient cohort. We used Pearson chi-square test and the independent *t* test to examine between-group differences in criteria with respect to the presence or absence of a poor 6MWT performance.

We constructed receiver-operating characteristic (ROC) curves, with area under the curve (AUC) data used to determine the test accuracy alone, or combined with covariates. Separate analyses were conducted for continuous and ordinal data. According to previous authors,^[23]^ an AUC >0.7 was used as the criterion of good discrimination.

To determine the combination score of each test and covariates, we calculate the predicted probability of being able to discriminate between a normal and poor 6MWT test score using logistic regression models (with enter method), where the dependent variable was a poor 6MWT (yes/no), and independent variables were each test and their covariates. To select these covariates, we initially explored among controls (i.e., patients with good 6MWT) the association between all measured covariates, and each marker, by means of linear regression (with enter method).^[24]^ Those covariates found to be significant were then selected.

In addition, we selected the best cut-off score for each test, when used alone. This was the value at which sensitivity + specificity − 1 was maximized. Using these cut-off scores, we calculated the sensitivity (Se), specificity (Sp), positive (LR+), and negative (LR−) likelihood ratios. We also calculated Se, Sp, and LRs for the combined scores, by examining data fit with regression models and predicted probabilities.

Finally, multivariate logistic regression analyses were used to assess the relative importance of the 2 tests (4MGS and 5STS) as clinical determinants of a poor 6MWT performance. Separate models were devised for each test in isolation, or combined with covariates. Each model was adjusted according to those clinical determinants of a poor 6MWT, as identified in the literature,^[7]^ which showed significant between-group differences (*P* < 0.10) in previous bivariate analyses. “Goodness-of-fit” and regression diagnostics for each model were assessed using methods as described elsewhere.^[24]^

As a rule of thumb, we tested 15 subjects per predictor; this was an estimate of the sample size needed to generate reliable data.^[25]^ We recruited a minimum of 120 participants, assuming a maximum of 8 predictors. All analyses were performed with the SPSS statistical software program (SPSS version 19.0; IBM SPSS, Chicago, IL).

## 3 Results

### 3.1 Characteristics of patients

A total of 173 patients with COPD were screened during the study period. Of them, 147 were initially enrolled as potentially eligible. All agreed to participate in the study, but 10 failed to meet our eligibility criteria. Therefore, 137 patients were finally included in our patient cohort. Participants’ characteristics are described in Table 1. Patients were generally male, with moderate to severe COPD. Of our cohort, 35.8% scored grade 2, or higher, on the mMRC scale, with 19% reporting symptoms of depression (HADS ≥8). Three values were missing in our 6MWT dataset (2.18%).

**Table 1:**
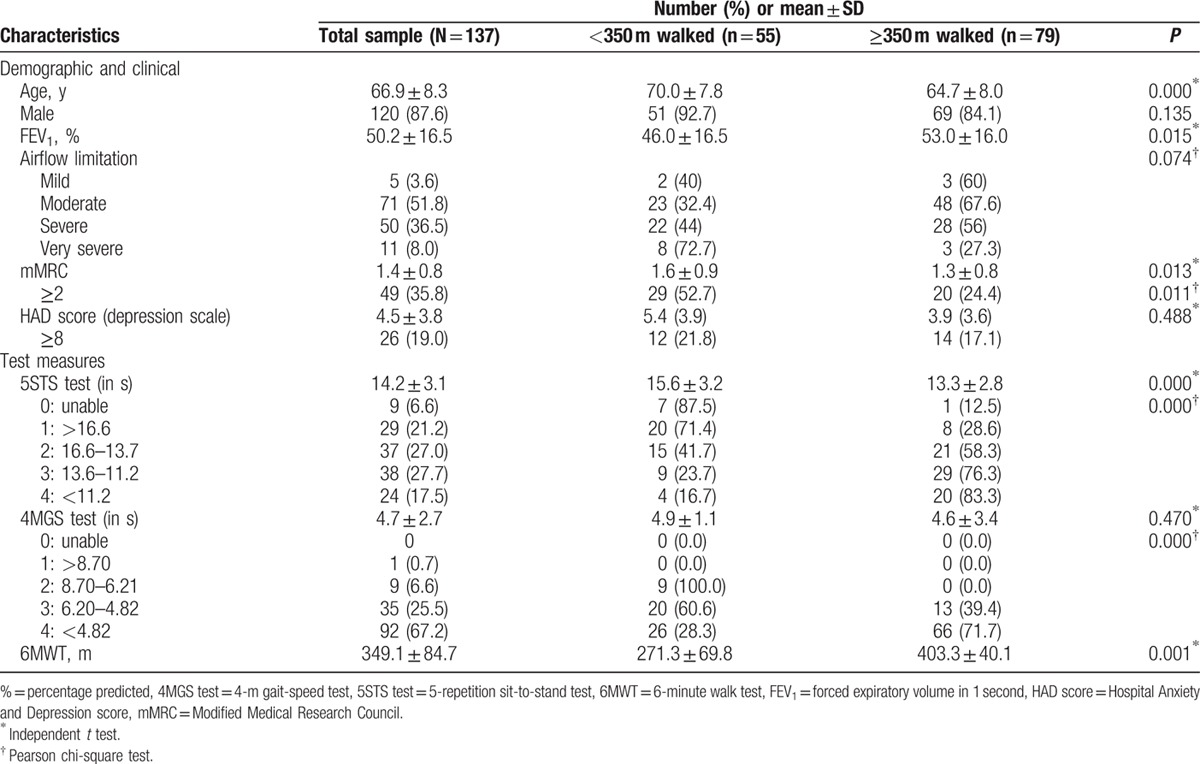
Patient characteristics stratified according to poor 6MWT performance.

A total of 55 patients (41.05%) had a 6MWT <350 m (mean 271.3 ± 69.8), with the remaining 79 patients (58.95%) demonstrating a 6MWT of ≥350 m (mean 403.3 ± 40.1). Participants with poor 6MWT performance were older, with a higher proportion of mMRC scores of 2, or higher (Table 1). Additionally, patients with poor 6MWT test results had lower mean FEV_1_ scores. No significant differences were found with the inclusion of depression as a covariate.

### 3.2 Discriminatory accuracy of the tests

**Figure 1:**
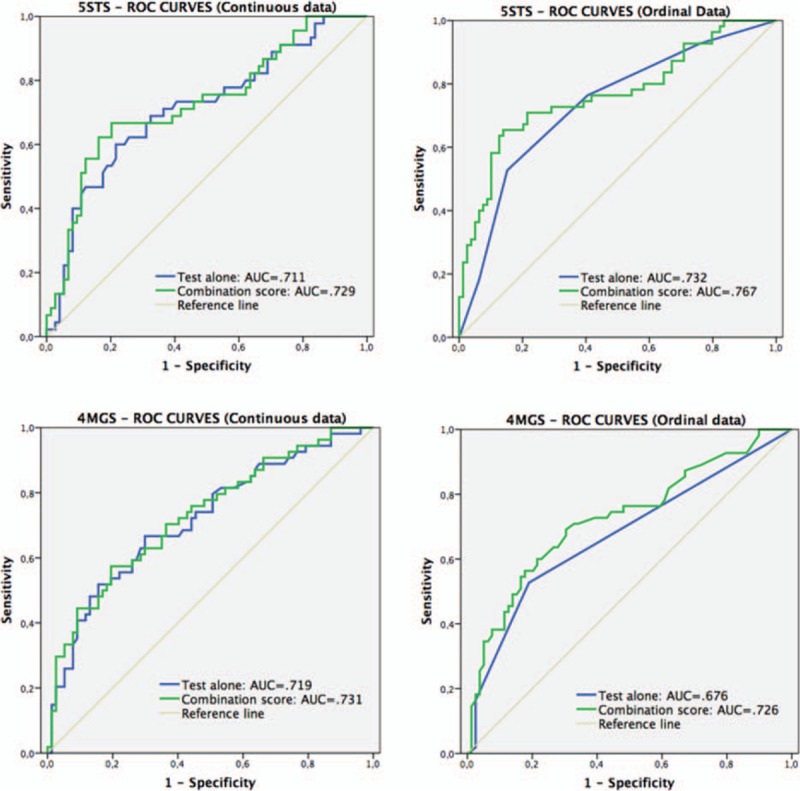
Receiver-operating characteristic curves comparing the 5STS and 4MGS tests alone (using continuous and ordinal data), with the predicted probability that combines each test and its covariates, in determining poor exercise tolerance. 4MGS test = 4-m gait-speed test, 5STS test = 5-repetition sit-to-stand test.

The ROC curves (Fig. 1) illustrated that the AUC for the 5STS test alone (in seconds) was 0.711 (95% confidence interval [CI] 0.613–0.809), and, when combined with significant covariates (age, CAT score, and QMS), increased slightly to 0.729 (95% CI 0.632–0.825). Using ordinal data, the AUC increased slightly from 0.732 (95% CI 0.645–0.819) for the test alone, to 0.767 (95% CI 0.681–0.852) for the “combination” score. The cut-off scores yielding the most accurate discrimination of a poor 6MWT using the test alone were 2 for ordinal measurements, and 13 seconds for continuous data. The values of Se, Sp, and LR, for these cut-offs, and combination scores, are shown in the Table 2.

**Table 2:**
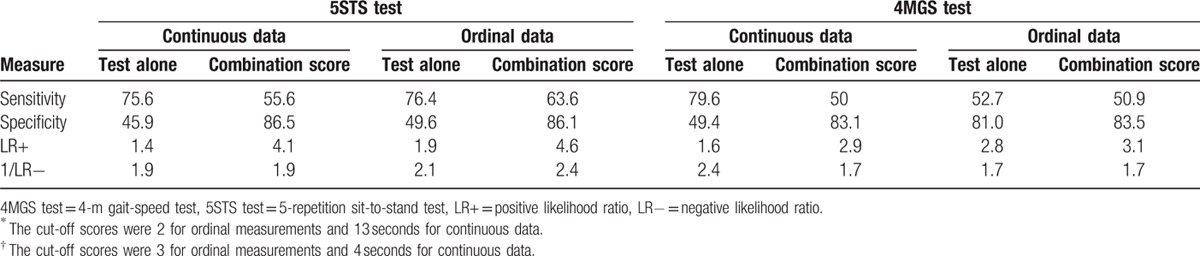
Accuracy of 5STS^∗^ and 4MGS^†^ scores, and of their predicted probabilities that combine each test and its covariates.

The AUC for the 4MGS test, when used alone (in seconds), was 0.719 (95% CI 0.629–0.809), and in combination, 0.731 (95% CI 0.643–0.819). Test accuracy was worse when using ordinal data, with an increase of accuracy >5% when using the combination score (using the covariates of age and symptoms of depression). The solo test yielded an AUC of 0.676 (65% CI 0.580–0.771), that is, poor, which increased to 0.726 (95% CI 0.637–0.816) in the combined score. The values of Se, Sp, and LRs are showed in Table 2 for cut-off scores of 3, and 4 seconds for ordinal and continuous data, respectively.

### 3.3 The value of each test as a clinical determinant

Results of multivariate models for determinants of a poor 6MWT performance are shown in Table 3. Model 1 shows that the 5STS test was a strong clinical determinant of a poor 6MWT, even after adjustment for other relevant clinical determinants. Using this model, only the 5STS test remained significant, recording an odds ratio (OR) of 1.23 (95% CI 1.05–1.44). This indicates that for every 1-second increase in the 5STS test result, a 23% increase (1–1.23) in the odds of recording a poor 6MWT performance is expected, as controlled for by other factors in the model. Model 2 shows that the 4MGS test was, by itself, not a significant predictor of performance. For this test, only the covariates of age and airflow limitation were retained.

**Table 3:**
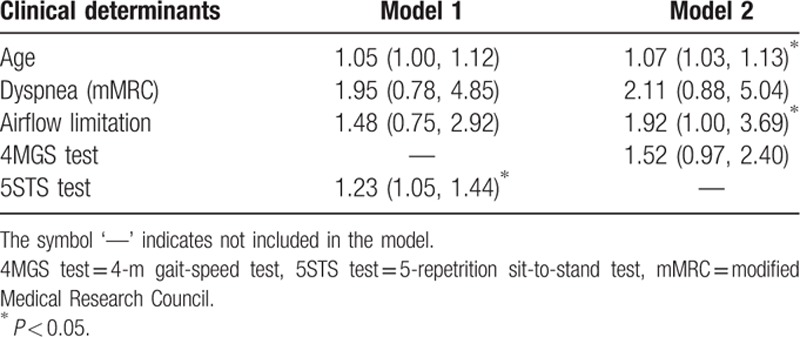
Adjusted multivariate logistic regression analysis of determinants of poor exercise tolerance.

## 4 Discussion

In this study, we examined whether the 5STS and 4MGS tests might be useful as markers and/or clinical determinants of poor exercise performance. We now report 2 important findings. First, continuous scores for the 4MGS and 5STS tests, and especially, ordinal scores for the 5STS test, were accurate tests with which to predict a poor 6MWT performance. Second, the 5STS test is a significant clinical determinant of poor performance (<350 m). To our knowledge, this is the first study to compare the accuracy with which these 2 tests can predict a poor 6MWT.

Our findings that ordinal scores of 2 or less collected using the 5STS test, and a time over 13 seconds or more, can usefully discriminate poor 6MWT performance, are novel in this field. Prior research has primarily used values based on anthropometric indicators^[26]^ and gait speed.^[8,11]^ In relation to the 4MGS test, our cut-off value of 4 seconds (or 1 m/s) is consistent with previous studies.^[27]^

The 4MGS test conducted using ordinal data could not accurately predict performance (AUC <0.7), although accuracy was strongly and positively affected by the inclusion of age and depression covariates. When, as in this case, the accuracy of a test depends on its covariates, it is commonplace to adjust for these variables in the statistical analyses.^[24]^ However, to our knowledge, this study is the first to assess the influence of covariates on test accuracy using the gait-speed test for COPD patients. Although adjustment for covariates is routine in other medical areas, this issue is still not well-appreciated for functional tests in COPD.

Our data showing that the accuracy of the 5STS test was unaffected by covariates, suggest that this test can be used alone, or adjusted. This finding has implications for situations in which one may choose to use the test alone, or use the combination score. When choosing between the 2 options, the duration of testing may be a factor. In settings where there are time constraints, a stand-alone test may be more practicable given that combination scores would necessitate QMS and CAT scores. On the contrary, discriminating between true and false cases may be a greater priority. It is important to note that our study showed that using the test alone, with ordinal data, resulted in higher Se and LR− values than the combination score, whereas the combination score generated higher Sp and LR+ values. Given these findings, the clinician should establish, before testing, the consequences (e.g., additional costs for care and rehabilitation) of diagnosing a poor 6MWT, for a patient with good exercise capacity, rather than a good performance for a patient with a poor 6MWT (where failure to initiate early intervention may be critical factor). So, for clinicians with an imperative to avoid false-negatives, the test alone may be more convenient.

The results of this study also showed that the 5STS test was a significant clinical determinant of poor 6MWT, unlike the 4MGS test. Our finding for the 4MGS test was unexpected given a previous study,^[11]^ which came to the opposite conclusion. A possible reason for the higher relevance of the 5STS test could be the extra respiratory demand required in this sit-to-stand test.^[10]^ Surprisingly, 3 clinical determinants identified by Spruit et al^[7]^ (age, airflow limitation, and dyspnea) were found to be insignificant in our model for the 5STS test. The closer approximation of this test to the 6MWT may explain the reduced influence of these pulmonary and nonpulmonary factors.

A valuable aspect of this study was its inclusion of covariates to determine the accuracy of testing. Although adjustment for covariates is common in therapeutic and etiologic studies, the issue of covariate effects has received little attention in the development of markers for screening or diagnosis.^[24]^ Our study now addresses this issue, and in doing so, has refined our thinking as to how to use functional tests in COPD patients. Our use of 2 kinds of data (ordinal and continuous) to explore the discriminatory accuracy of the 5STS and 4MGS tests is an additional strength in our approach, and the consistency of our results, using both kinds of data, reinforces our findings.

Our study had several limitations. First, our sample was recruited from a public Spanish hospital; our data may not apply equally to other settings. Therefore, until further research is conducted using a broader patient cohort, these results should be interpreted with caution. Second, we implemented a cross-sectional design in our study; our data must now be confirmed using additional prospective studies. Third, although the identified covariates for the 2 tests were relevant in terms of discriminatory accuracy, it may be speculated that other factors, not included in this study (e.g., disability, physical activity, body mass index), could also impact accuracy.

In summary, the present study shows that 5STS scores can be used as a robust clinical determinant of a poor 6MWT, and a marker with which to classify poor performance for COPD patients, especially when using ordinal data. The 4MGS test score alone is also a good marker when measured using continuous data. Covariate effects on accuracy were only relevant for the 4MGS test, when measured using ordinal data. This information could be useful for the selection and interpretation of these tests, so that appropriate care and/or preventative interventions can be provided. Further research should focus on whether changes in test data over time (using either test) can increase their usefulness.

## Acknowledgments

The authors wish to thank their patients, and the personnel of the hospital unit, for their cooperation during the course of this study.
